# RACIAL/ETHNIC GROUP DIFFERENCES IN OLDER ADULTS’ INVOLVEMENT WITH ADULT PROTECTIVE SERVICES

**DOI:** 10.1093/geroni/igae098.0269

**Published:** 2024-12-31

**Authors:** Kenneth Steinman

**Affiliations:** The Ohio State University, Columbus, Ohio, United States

## Abstract

Child protective services has long wrestled with concerns about Black and Hispanic families being disproportionately more likely to be investigated. In contrast, only one, small, recent study has quantified racial/ethnic group differences in adult protective services (APS). A paucity of research and inconsistent findings in this area have limited the generalizability of studies and complicated efforts to develop culturally sensitive practices for specific groups. The proposed project would represent the first national study of racial/ethnic group differences in older adults’ involvement in APS. It would provide the several key findings, including: a conceptual framework that explains why racial/ethnic group differences in APS may exist; benchmarks for the “typical” magnitude of racial/ethnic group differences in key APS metrics across most US states; identification of mistreatment types, genders, and age groups where racial/ethnic group differences in APS metrics are especially large; identification of the types of racial/ethnic group differences in APS metrics that vary markedly from state to state or county to county; and descriptions of the community (e.g., poverty rate) and organizational (e.g., APS agency) characteristics that help explain this variation. The project will also engage researchers, practitioners, and community leaders in three advisory teams. Their regular involvement will help ensure that the project employs the best available theories and methods, provides useful guidance for APS professionals, and reflects the priorities and lived experiences of the communities being studied.

